# Interactions of Lipid Droplets with the Intracellular Transport Machinery

**DOI:** 10.3390/ijms22052776

**Published:** 2021-03-09

**Authors:** Selma Yilmaz Dejgaard, John F. Presley

**Affiliations:** 1 Department of Anatomy and Cell Biology, McGill University, Montreal, QC H3A 0C7, Canada; selmayilmaz810@gmail.com; 2 Department of Medical Biology, Near East University, Nicosia 99138, Cyprus

**Keywords:** lipid droplet, biogenesis, endoplasmic reticulum, autophagy, microautophagy, mitochondria

## Abstract

Historically, studies of intracellular membrane trafficking have focused on the secretory and endocytic pathways and their major organelles. However, these pathways are also directly implicated in the biogenesis and function of other important intracellular organelles, the best studied of which are peroxisomes and lipid droplets. There is a large recent body of work on these organelles, which have resulted in the introduction of new paradigms regarding the roles of membrane trafficking organelles. In this review, we discuss the roles of membrane trafficking in the life cycle of lipid droplets. This includes the complementary roles of lipid phase separation and proteins in the biogenesis of lipid droplets from endoplasmic reticulum (ER) membranes, and the attachment of mature lipid droplets to membranes by lipidic bridges and by more conventional protein tethers. We also discuss the catabolism of neutral lipids, which in part results from the interaction of lipid droplets with cytosolic molecules, but with important roles for both macroautophagy and microautophagy. Finally, we address their eventual demise, which involves interactions with the autophagocytotic machinery. We pay particular attention to the roles of small GTPases, particularly Rab18, in these processes.

## 1. Introduction

The study of intracellular membrane trafficking has historically concentrated on the movement of proteins and lipids between the membrane-bound compartments of the secretory and endocytic pathways. More recently, relationships have been found between other organelles and membrane trafficking pathways, including melanosomes [1], peroxisomes [2] and lipid droplets. While historically lipid droplets were considered inert storage structures, increasing evidence suggests that they were highly dynamic structures which interacted with numerous intracellular organelles, including endoplasmic reticulum (ER), endosomes, plasma membrane and mitochondria. Interestingly, some of these functions are regulated by families of proteins known to be important in membrane trafficking pathways, including ADP ribosylation factors (Arfs), Rabs and tethers.

The existence of adipocytes as a major stores of lipids has been known since the mid-19th century (reviewed in [3]). The body of white adipocytes is primarily made of a single large lipid droplet (LD), which takes up most of the cytoplasm, pushing the nucleus and other organelles to the side, while brown adipocytes contain multiple smaller lipid droplets [3]. Subsequently, LDs were found in a large variety of cell types, including hepatocytes [4]. Fat globules in milk were found to originate as LDs [5]. LDs were also found in some cell types under pathological conditions. Most notably, foam cells, found in artery walls at an early stage in the development of atheroschlerotic lesions, were found to be macrophages and smooth muscle cells with a large portion of their cytoplasm taken up by LDs subsequent to massive uptake of lipoprotein particles [6].

LDs were originally considered to be static stores of triglycerides and cholesterol esters. However, they are now known to be dynamic organelles which interact extensively with other organelles and numerous regulatory pathways to support the logistics of lipid storage and mobilization. This story was fleshed out with the identification of enzymes, which produced neutral lipids, primarily in the ER, and cytoplasmic lipolytic enzymes which could bind to the surface of the LD. Direct interactions have been found between LDs and other organelles, including notably the ER and mitochondria, which are the organelles with the greatest need for stored lipids. These interactions are regulated by a still poorly understood network of proteins, including tethers and Rabs such as Rab18 that are also found on elements of the secretory and endocytic pathways. More recently, a significant role in autophagy of LDs (lipophagy) has been found for lipolysis of LDs, acting in parallel to cytoplasmic lipases. This review will focus on the interactions between LDs and the secretory and endocytic pathways at different stages of their life cycle, from birth to death, and on commonalities between LDs and the more conventional membrane-bilayer-bound organelles that make up the secretory and endocytic pathways. As a number of distinct topics are covered and the total volume of relevant literature is large, this review is not intended to be comprehensive, but instead to give a broad overview, with a focus on recent findings.

## 2. Biogenesis of Lipid Droplets from ER Membranes

There is considerable evidence that LDs arise from the ER in a wide variety of eukaryotic organisms as diverse as mammals, higher plants and yeast. While exit of proteins from the ER via the secretory pathway has been intensively studied, the comparable process of LD production from the ER is still incompletely understood. We will begin by briefly comparing the two processes.

**Figure 1:**
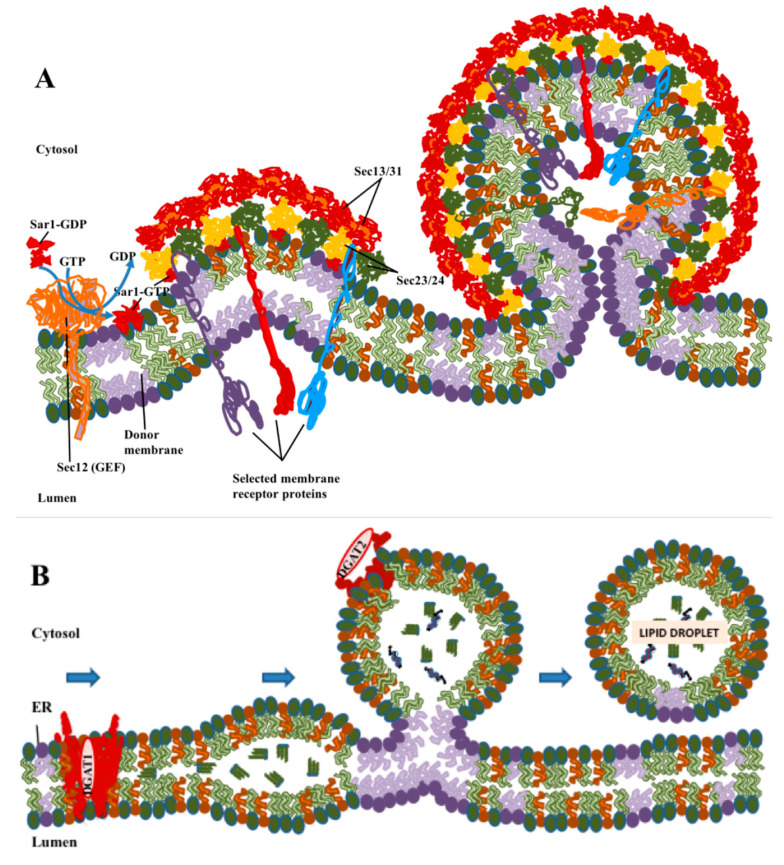
Comparison of the formation of coatomer protein II (COPII) vesicles with biogenesis of LDs. (**A**) COPII polymerizes laterally to form coated buds, with cargo proteins being sorted into the vesicle through interactions of their cytoplasmic domains with the COPII coat. Coat assembly and sorting take place primarily through protein–protein interactions. (**B**) Lipid droplet (LD) formation begins with the synthesis of neutral lipids: triglycerides by diacylglycerol O-acetyltransferase 1 (DGAT1) or cholesterol by acetyl-CoA acetyltransferase (ACAT)1/2. Neutral lipids condense into lenses in ER membranes, which then bud to form LDs. DGAT2 and other LD-resident proteins with short hydrophobic loops that are stable in a lipid monolayer can move to LDs while still attached to ER. These processes are driven primarily by phase separation and biophysical processes of lipids, but with proteins playing important modulatory roles.

Exit of integral membrane proteins from the ER occurs in specialized domains found on the surface of the ER, that are termed ER exit sites or transitional elements [7,8]. Coat protein complex II (COPII) [9] binds to the surface of the ER exit site to form domains which can trap cargo proteins (Figure 1A), typically through interactions between motifs on the cytoplasmic domain of the protein and the COPII protein [10]. This was originally thought to lead to the formation of a detached COPII vesicle of about 80 nm in diameter, which then delivers the cargo to a structure termed a vesicular-tubular complex (VTC), which forms near the ER exit site and is capable of carrying the cargo to the Golgi apparatus using the microtubule motor dynein [9,11,12]. More recent results, including studies in yeast [13], and studies of large cargoes such as procollagen [14,15] and very low density lipoproteins (VLDL) [16] too large to fit in COPII vesicles, have led to variations on this model, including evidence for more flexible polymerization of COPII to form larger non-spherical transport intermediates into which procollagen can fit [17]. An additional motivator for new models is that it proved difficult to easily identify COPII vesicles in intact cells by electron microscopy [13]. Kurokawa proposed, based in part on work in yeast, that COPII concentrates cargo in domains on the ER that included coated buds, but that delivery of cargo from these domains to the cis face of the Golgi occurs through a kiss-and-run mechanism (reviewed in [18]). However, despite the differences between these models of COPII trafficking, all agree that cargo is primarily concentrated in COPII-coated domains as the result of interactions between protein molecules.

LDs are organized differently to conventional membrane-bound organelles in that their surface is covered by a lipid monolayer rather than a bilayer (Figure 1B), and their interior consists of hydrophobic neutral lipids rather than an aqueous lumen. Although fully transmembrane proteins cannot enter the LD, integral membrane proteins anchored by hairpins that pass only through the outer leaflet are stable in this monolayer [19]. In contrast to the formation of COPII vesicles, the roles of protein in LD formation, while important, appears to be more limited. The major neutral lipids are triglycerides in which the final synthesis step is carried out by the enzymes diacylglycerol O-acetyltransferase 1 (DGAT1) [20] and diacylglycerol O-acetyltransferase 2 (DGAT2) [21], and cholesterol esters, synthesized by acetyl CoA acetyltransferase 1 (ACAT1) [22] and acetyl CoA acetyltransferase 2 (ACAT2) [23]. Of these enzymes, three, DGAT1, ACAT1 and ACAT2, are multipass membrane-spanning proteins which require the presence of a conventional membrane bilayer and reside primarily in the ER. DGAT2 is also an integral membrane protein but is anchored by a hairpin loop [24], which in principle allows for the presence of the protein molecule in either a membrane bilayer or monolayer. DGAT2 is found in the ER, but, as will be discussed later, it can also be directly anchored in the phospholipid monolayer found at the surface of LDs [19].

The presence of these enzymes indicated that the ER is a major site of neutral lipid synthesis. Computer simulations and biophysical considerations suggested that neutral lipids produced in the bilayer of ER membranes would remain soluble in the inner hydrophobic portion of the membrane bilayer till a concentration of approximately 3% is reached [25]. At higher concentrations, neutral lipids would begin to condense and form lenses [25]. These could then grow by acquisition of additional excess neutral lipid as it was synthesized, or by fusion should two lenses encounter each other [25]. Biophysical analysis indicated that in principle, given the right conditions, separation of a lens from the ER to form an LD could proceed spontaneously due to achieving a lower energy state [26].

These biophysically based hypotheses made a number of predictions which have been verified experimentally. Lens formation could be visualized by electron microscopy of giant unilamellar vesicles [27] or in the ER of cells [28]. However, the fact that in cells LDs bud exclusively into the cytoplasm and not the ER, requires an explanation. In principle, if the ER bilayer were symmetric and LD formation were determined purely by biophysics of the lipid species involved, LD budding would be expected to be random, with half of new LDs budding into the ER lumen, which is not observed in normal cells. Therefore, the asymmetric budding of LDs into the cytoplasm that is actually observed must result either from asymmetric distributions of lipids in the ER bilayer or from the actions of proteins (not taken into account in this simple model).

**Figure 2:**
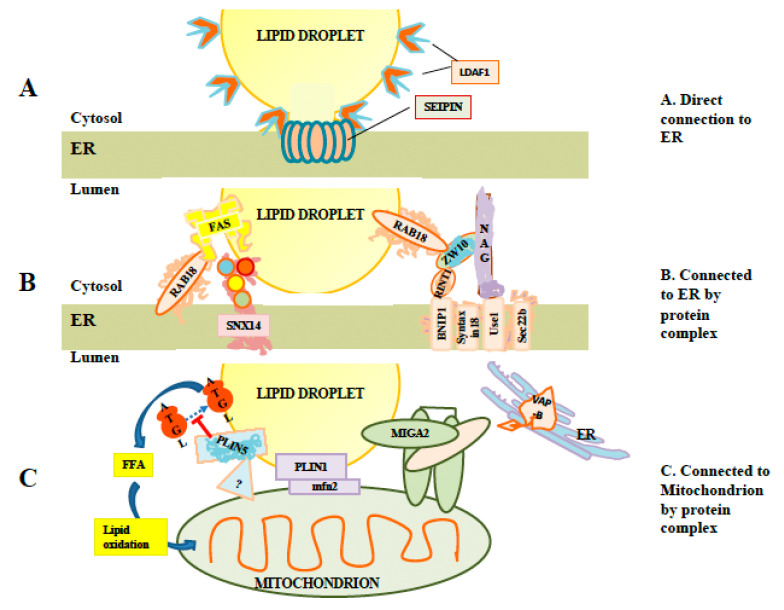
Interaction of LDs with other membranes. (**A**) Illustration of a direct connection. LDs can remain attached to the ER by direct membranous connections stabilized by seipin rings. These connections permit exchange of proteins such as lipid droplet assembly factor 1 (LDAF1) between LDs and ER membranes. (**B**) Examples of protein complexes which may tether LDs to ER in the absence of direct membranous connections, including SNX14 and the Rab18/NRZ complex. While Rab18 is required for recruitment of fatty acid synthase to LDs, the link representing binding between fatty acid synthetase (FAS) and Rab18 is speculative. (**C**) Examples of proteins and protein complexes tethering LDs to mitochondria. At least two perilipins (PLIN1 and PLIN5) are involved. The mitoguardin 2 (MIGA2) complex may be involved in forming three-way complexes involving mitochondria and LDs linked to ER via VAP-B.

Interestingly, it has been reported that LD budding is primarily from tubular rather than flat ER membranes [29,30]. Tubulation of membranes favors asymmetric distribution of lipids between the inner and outer layers of ER membranes [29,30]. Furthermore, there is evidence of the involvement of a number of proteins in LD formation (reviewed in [31]). Seipin is one protein which has been particularly studied. Mutation of seipin impacts LD formation in mammalian cells, affecting the size distribution of LDs, with a small number of LDs being abnormally large [32]. The exact role of seipin is unclear. However, it has numerous interaction partners, which have been linked to lipid metabolism or LD formation, including glycerol-3-phosphate acyltranserase 3 (GPAT3), glycerol-3-phosphate acyltransferase 4, (GPAT4), receptor expression-enhancing protein 1 (REEP1), 14-3-3B, sarco/endoplasmic reticulum Ca2^+-^ATPase (SERCA), stearoyl-CoA desaturase-1 (SCD1) and Ldo (reviewed in [31]), suggesting a possible organizational role. Seipin is an integral membrane protein with two transmembrane domains. Cryo-EM analysis has shown that seipin subunits organize into oligomeric ring-shaped structures [33]. It has been suggested that these could be involved in lipidic bridges [19] that appear to exist between ER and budded LDs [32] and could control access of ER proteins and lipids to the LD [32] (illustrated in Figure 2A).

In yeast, deletion of seipin, together with the reticulon-like protein Pex30, results in a strong block of LD production, while knockdown of Pex30 alone has little effect [34]. Interestingly, the double knockdown also blocks budding of peroxisomes from ER membranes. This could suggest some sharing of elements between the peroxisome budding machinery and LD budding. This is surprising since peroxisomes are conventional membrane-bound organelles with an aqueous lumen surrounded by membrane bilayers, and are thus very different from LDs. One explanation is that peroxisomes and LDs have similar need to shape ER membranes during the budding process, and that seipin or proteins recruited by seipin play a role in this process. Chung [35] reported that seipin recruits large, possibly stoichiometric, amounts of lipid droplet assembly factor 1 (LDAF1) to ER foci, followed by the rapid appearance of triglycerides and the perilipin family member perilipin 3 (PLIN3). Subsequently, LDAF1 lost its association with seipin and migrated onto the surface of the nascent LD [35] (Figure 2A). They hypothesized that hydrophobic regions of LDAF1 formed two hairpins through ER membranes, and that the association of LDAF1 with seipin rings would lead to the rapid colocalization of large numbers of protein hydrophobic domains, forming a domain favorable to the accumulation of triglycerides. This contrasts with an alternate view in which seipin puncta capture pre-existing triglyceride lenses [36]. Recent molecular dynamic simulations have supported the idea that the tightly clustered hydrophobic domains in seipin clusters can nucleate triglyceride lenses [37].

Fat storage inducting transmembrane protein 2 (FIT2)/Scs3 is another protein which plays a key role in LD formation [38]. Knockdown of Scs3, the yeast homologue of FIT2, leads to the formation of aberrant LDs which remain embedded in ER membranes [28]. This suggests a requirement for FIT2 in LD budding. FIT2/Scs3 may control diacylglycerol (DAG) levels at LD budding sites. In one proposal, lens formation is favored by local production of DAG, which favors negative membrane curvature [39]. Local reduction of DAG levels by FIT2 could destabilize the lens and favor budding.

Perilipin family proteins may also favor stabilization of LD lenses or LD budding. Perilipins are a family of cytoplasmic proteins [40] (perilipin 1(PLIN1)/perilipin, perilipin 2 (PLIN2)/adipose differentiation related protein (ADRP), PLIN3/Tip47, perilipin 4 (PLIN4) and perilipin 5 (PLIN5)) which have domains that can target them to the LD surface [41] and which act as important scaffolding/coat proteins on the LD surface. There is some evidence that perilipins bind to and stabilize ER-localized lipid lenses and favor LD formation [42]. Knockout of one perilipin, PLIN3/Tip47, in mammalian cells was insufficient to block LD budding by itself, but subsequent inhibition of the small GTPase Arf1 with brefeldin A, together with siRNA knockdown of PLIN3, completely blocked LD budding [43]. This suggests a role for some perilipins in LD formation at least for subpopulations of LDs in some cell types.

Overall, while exit of proteins from ER via the COPII-mediated pathway appears to rely primarily on protein–protein interactions, with a possible ancillary role for lipid partitioning, formation of LDs from ER membranes depend primarily on biophysical considerations, with proteins playing important but ancillary roles.

## 3. Growth of LDs and the Roles of Interactions with Other Intracellular Membranes

LDs continue to grow after formation from ER membranes, sometimes to a significant size. This appears to take place via several mechanisms including continued direct connections to ER via membrane stalks to allow passive movement of neutral lipids, action of lipid exchange proteins at ER-LD contacts, and synthesis of triglycerides by DGAT2 anchored to the LD monolayer. There is increasing evidence of a variety of connections between LDs and other organelles.

### 3.1. Direct Connections to Endoplasmic Reticulum

Budding from the ER is often incomplete, with stalks continuing to connect nascent LDs to the ER, e.g., [32,36,44]. A light microscopy-based analysis has indicated that in some cells, as many as 85% of LDs may maintain lipid-based connections to ER [45]. Seipin rings have been proposed to stabilize these links [32] (Figure 2A). A significant subset of LD-localized proteins, including LDAF1, DGAT2, and glycerol-3-phosphate acyltransferase 4 (GPAT4), are membrane-localized by hairpins which extend through only a single leaflet of the ER bilayer [19]. Such hairpins would be stable also in the lipid monolayer surrounding the LD.

There is evidence from photobleach experiments that GFP-tagged DGAT2 and LGPAT can move freely between LDs and ER membranes. In one study, GFP-LGPAT membranes recovered after photobleach of the LD for up to eight hours after LD formation, suggesting prolonged connection of the LD with ER membranes in COS7 cells [19]. However, at later times, connection with ER membranes was lost. A second study from the same group argued that de novo formation of membrane bridges with preexisting LDs was possible and depended on the adjustment of membrane tension on the LD through modification of the LD monolayer by coatomer protein I (COPI)/Arf1 [46]. In their model, COPI and Arf1 bud coated “nanodroplets” from the LD surface while also selectively removing phospholipids from the surface monolayer, resulting in changes in surface tension favorable to the formation of membrane bridges to ER. Thus, disconnection of a LD from ER would be a reversible process, and reconnection could occur under certain conditions.

DGAT2 is one integral membrane protein which has been reported to be anchored to LDs by a hairpin which goes no further than halfway through the ER bilayer [24], and as such can also rest within a membrane monolayer such as those that are found on the surface of LDs. DGAT2 has been shown to be both on the surface of LDs and in ER membranes. It could be sequestered on the LD surface after budding but has also been argued to diffuse between the ER and LDs via membrane bridges [19]. In situ synthesis of neutral lipids by enzymes resident on the LD membrane including DGAT2 could potentially prevent their accumulation in ER, reducing toxicity and ER stress in situations where LDs already existed.

### 3.2. Protein Tethers between Lipid Droplet and Endoplasmic Reticulum Membranes

LDs can tether to the ER via protein complexes independently of any stalk or bridge, as evidenced by electron-rich densities often appearing between LDs and ER [47] and by the more recent identification of at least some of the complexes involved. Furthermore, electron microscopy often shows LDs directly opposed to ER, with prolonged regions of contact between the LD and ER, or even wrapping of ER around the LD [48]. In principle, close proximity can facilitate lipid transfer between the two membranes, including transfer of neutral lipids and free fatty acids. Similarly, LDs frequently contact other intracellular organelles, including most notably mitochondria and peroxisomes, both of which are important as consumers of stored lipids. While protein connections were initially suspected based primarily on the basis of density appearing in EM images, a number of the protein complexes involved have been identified and partially characterized. These are described below. Selected examples are shown in Figure 2B.

### 3.3. Rab18/NRZ Tethering Complex

The small Ras-family GTPase Rab18, was reported to drive association of LDs with ER membranes in overexpression experiments, with an association being reported, both using light microscopic and electron microscopic assays [48]. The same study found Rab18 on a subset of LDs lacking PLIN2/ADRP. Martin [49] also reported the heterogeneous appearance of Rab18 on LDs, with new LDs induced by oleate addition lacking Rab18, and acquisition of Rab18 on a subset of LDs beginning about three hours after LD induction. Rab18 containing LDs were found to show a reciprocal association with caveolin 3, which was found solely on LDs lacking Rab18. Rab18 association with LDs could be increased by inducing lipolysis, while blocking lipolysis abrogated Rab18 association [49]. These data suggest a post-biogenesis role for Rab18 on LD membranes. Supporting this, Xu [50] reported that knockdown of Rab18 did not impair the formation of LDs, but that 100 nm diameter LDs accumulated and then failed to expand. However, Jayson [51] reported that knockdown of Rab18 failed to produce an LD phenotype. As Rab40c shares sequence similarity to Rab18 which may result from a gene duplication event [52], one explanation is that some Rab18 functions are redundant with other proteins, which could result in cell-type specific masking of the Rab18 phenotype.

Rab18 association with ER (Figure 2B) was subsequently reported to be mediated by the NRZ tethering complex [50]. This complex is composed of several subunits including neuroblastoma-amplified gene (NAG), zeste white 10 (ZW10) and RAD50 interactor-1 (RINT1). ZW10 is proposed to bind to LD-associated Rab18, and with the NAG subunit, which in turn binds to the integral membrane synaptosomal-associated protein receptor (SNARE) protein Use1. Use1 is embedded in ER membranes in a complex together with BN1P1, Sec22b and the SNARE Syntaxin18 [50]. Interestingly, the SNAREs involved appear to also play a role in ER/Golgi trafficking [53]. Whether these SNAREs are simply an inert part of a tethering complex or whether they can instead drive hemifusion with the LD is currently unknown. To drive hemifusion, SNAREs would need to be on LDs as well, but there is some evidence that this is the case, as knockdown of the genes for the SNAREs synaptosomal-associated protein 23 (SNAP23) and vesicle-associated membrane protein 4 (VAMP4) have been reported to decrease LD-LD fusion [54]. Recruitment of fatty acid synthase (FAS), which synthesizes fatty acids including palmitate, to LDs by Rab18 has been reported [55]. Although FAS recruitment was reported under conditions of dengue virus infection, it required Rab18 to be GTP-associated [55], consistent with a role for FAS as a Rab18 effector. Recent work has also implicated Double FYVE-containing protein 1 (DFCP1) as a possible Rab18 effector or interactor. DFCP1 was reported to relocate from a variety of other organelles to LDs after oleic acid (OA) addition via a mechanism independent of the FYVE domains and to interact with Rab18 [56]. In that study, overexpression of DFCP1 increased LD size while decreasing total LD number. Another study [57] also implicated an association between DFCP1 and Rab18 and a role for DFCP1 in promoting LD growth. This study reported that this association required Rab18-GTP, consistent with DFCP1 being a Rab18 effector. They also found Rab18-dependent association of DFCP1 with ZW10. In this study, DFCP1 formed puncta, which was associated with nascent LDs, and data from EM thin sections indicated that DFCP1 enhanced the association between ER membrane and mature LDs [57]. Overall, these data potentially implicate DFCP1 as a Rab18 effector with multiple roles, both in tethering and in LD biogenesis.

### 3.4. Rab40c

A gene duplication event prior to formation of the vertebrate lineage created first Rab40, and then Rab40a, Rab40b and Rab40c from Rab18 through a series of gene duplications [52]. There is no evidence to date for a role of Rab40a and Rab40b on LDs. However, Rab40c has been reported on LDs, and deletion of Rab40c or overexpression of its GTPase activating protein (GAP) disabled homologue 2 interacting protein (DAB2IP) caused accumulation of LDs [58,59]. While the role of Rab40c in LDs is unknown, there are reports linking it to degradation of other proteins. It regulates ubiquitination of receptor of activated protein C kinase 1 (RACK1) [60], and proteosomal degradation of vacuolar protein sorting 9-domain-containing protein (VARP) [61]. VARP [62] and Rab40c have also been linked with recycling endosomes involved in myelin formation [63]. Given the paucity of data, the role of Rab40c is unclear, but its relationship to endosomes and to protein degradation could be consistent with a role in the autophagy of LDs.

### 3.5. Snx14 (Mdm1)

Snx14 and its yeast homologue, Mdm1, are members of the sorting nexin family of proteins found on ER membranes. Hariri [64] reported that Mdm1 was localized to regions of the yeast nuclear envelope adjacent to the vacuole, and that it recruited enzymes involved in fatty acid synthesis to these regions including at least the fatty acid CoA ligase Faa1. New LDs were formed at these contact sites. Deletion of MDM1 resulted in a block in neutral lipid synthesis and a greatly decreased number of LDs forming. This suggested a role for MDM1 in LD biogenesis in yeast. In contrast, Snx14 [65] was visualized in electron micrographs to be at contact sites between LDs and ER in mammalian cells after loading with OA (Figure 2B). This association was unaffected by deletion of seipin. Deletion of Snx14 did not appear to reduce biogenesis of LDs, but to impair growth of LDs after their formation, without affecting overall cellular levels of neutral lipids. This study proposed that Snx14 was an integral membrane ER protein, binding the LD lipid monolayer in trans to stabilize the ER/LD contact and to facilitate lipid transfer to growing LDs. Whether it associates with other protein complexes is unknown [65].

### 3.6. Lipid Transfer Proteins

The ORP (OSBP Related Protein) family of oxysterol binding proteins may play a role in sterol and phospholipid transfer between adjacent membranes. Two members of this family, ORP5 and ORP8, are integral membrane proteins localized to the ER (reviewed in [66]). Du et al. [67] reported that mCherry-ORP5 dynamically localizes to contacts between ER and LD and proposed that it transports phosphatidylserine between ER and LDs. Deletion of ORP5 results in an increase in mean LD size. ORP2 [68] is a sterol-binding protein which can associate with several organelles, including LDs. ORP2 has been reported to interact with both phospholipids and cholesterol [69], so it could potentially play a role in shuttling cholesterol to LDs. However, ACAT1 and ACAT2, multipass membrane-spanning proteins which esterify cholesterol, are found only in ER membranes. A recent study [70] reported that ORP2 binds adipose triglyceride lipase (ATGL) and the beta subunit of COPI, which could suggest an alternate role for ORP2 as a scaffold, facilitates the recruitment of other proteins to the LD surface. Another family of lipid transfer proteins implicated in contacts between ER and LDs are vacuolar protein sorting 13A (Vps13A) and vacuolar protein sorting 13C (Vps13C), proteins initially shown to be involved in trafficking to the vacuole in yeast with strong structural similarities to Atg2 (reviewed in [71]). Kumar and coworkers [72] reported that Vps13A tethered ER to mitochondria, while Vps13C tethered ER to late endosomes/lysosomes. In their study, both Vps13A and Vps13C tethered ER to LDs, and they also reported that an N-terminal portion of these proteins could transfer NBD-phosphatidylserine between membranes. Their functional significance is unknown. However, VPS13A was reported to influence motility of LDs by Yeshaw and coworkers [73], and VPS13C was reported to inhibit lipolysis in brown fat adipocytes [74].

### 3.7. Interactions with Mitochondria

The perilipin family member PLIN5, which is localized on the surface of LDs in cells such as skeletal muscle which have high energy demands, has been reported to tether LDs to mitochondria [75,76,77,78], helping to establish a metabolic linkage between the organelles. PLIN5 has recently been reported to be localized to LD-mitochondrial junctions by super-resolution microscopy, and to be absent from other regions of the LD surface [76]. As PLIN5 has also been shown to interact with ATGL [79], in addition to tethering LDs to mitochondria, it may also localize lipolysis machinery to the LD-mitochondrial interface to facilitate the transfer of free fatty acid to the mitochondrion. In contrast, perilipin family member PLIN2 was excluded from mitochondrial junctions in the same study. However, PLIN1 was reported by Boutant [80] to bind LDs to the mitochondrial protein Mfn2 in brown fat tissue. Mfn2 is known to be involved in homotypic fusion of mitochondria but had not previously been known to mediate interactions between mitochondria and LDs. Another protein, mitoguardin 2 (MIGA2), has been implicated in linking the ER, LDs and mitochondria [81]. These interactions are summarized in Figure 2C. As with ER/LD interactions, it is likely that there are other unknown factors and complexes associating the LD membrane with mitochondria. The known interactions discussed here are illustrated in Figure 2C.

### 3.8. Interactions with Peroxisomes

While LDs often appear to be associated with peroxisomes by electron microscopy, the nature of these associations is not well understood. However, recent work has identified some proteins that appear to link LDs to peroxisomes. Chang et al. [82] reported that M1 Spastin on LDs, a protein previously associated with genetic neurological disorders and microtubule severing [83] links LDs to the fatty acid transporter adenosine triphosphate binding cassette subfamily D member 1 (ABCD1) found on peroxisomes. In a separate study, Kong et al. [84] found a role for the peroxisomal protein peroxisomal biogenesis factor 5 (PEX5) in the formation of contacts between peroxisomes and LDs. They also proposed that PEX5 aids in recruiting the lipolytic enzyme ATGL to LD/peroxisome contact sites. More research will be needed to determine if these proteins have related functions or whether there are multiple independently regulated tethering complexes which could mediate peroxisome/LD interactions.

## 4. Catabolism of Lipid Droplets by Cytoplasmic Lipases and Lipophagy

Catabolism of LDs can take place through the action of cytoplasmic lipases, which can bind to the LD surface, or through interactions with the endocytic pathway including macroautophagy (lipophagy) and microautophagy.

### 4.1. Cytoplasmic Lipases

**Figure 3:**
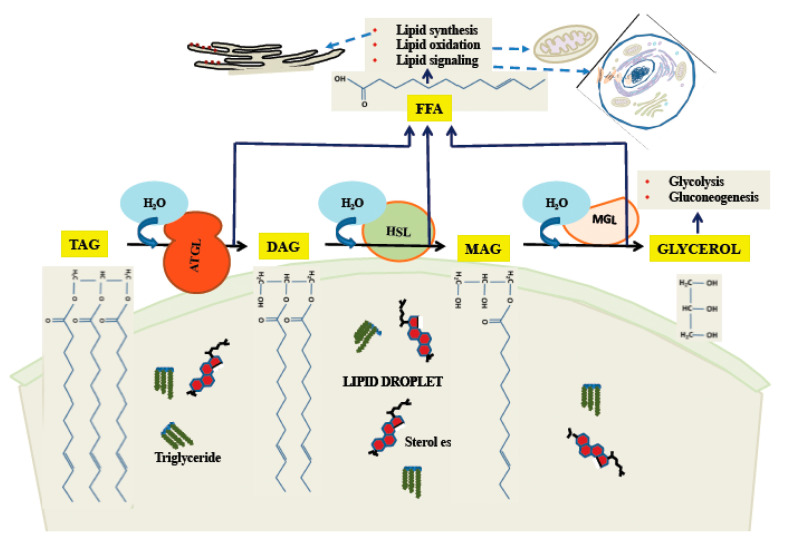
Triglyceride catabolism by cytoplasmic lipases on the LD surface. ATGL catalyzes release of the first fatty acid, followed by HSL and MAG in sequence. While ATGL and MAG seem to have narrow specificity as shown here, HSL has some activity towards TAG.

One important means by which stored lipids can be released is through the action of cytoplasmic lipases. As the body of the LD is primarily made of triglycerides, release of free fatty acid (FFA), which can be used for energy, and glycerol requires three hydrolytic steps. It is primarily the cytoplasmic enzyme ATGL [85] which initiates the first step, converting a triglyceride molecule to DAG and one FFA [86]. Hormone-sensitive lipase (HSL) releases the second FFA [87], although it can initiate the process as well [88], albeit less efficiently than ATGL. The third enzyme is monoglyceride lipase (MGL), which has as its products FFA and glycerol [89]. The process of release of FFA and glycerol from neutral lipids is illustrated in Figure 3. The regulation of these enzymes can occur by a variety of pathways, the best understood of which is the activation of HSL by a pathway involving cyclic adenosine monophosphate (cAMP) and Protein Kinase A (PKA; reviewed in [90]). In this pathway [91], the G-protein-coupled β-adrenergic receptor, upon binding ligand, activates cAMP production, which leads to activation of PKA. PKA phosphorylates cytoplasmic HSL, activating it and causing it to bind to perilipins on the LD surface, leading to increased catabolism of neutral lipids. While the PKA/cAMP pathway was the first to be defined, a large variety of other pathways exist, forming a complex network regulating all of the major degradative enzymes. For a recent review, see [92].

### 4.2. Macroautophagy

**Figure 4:**
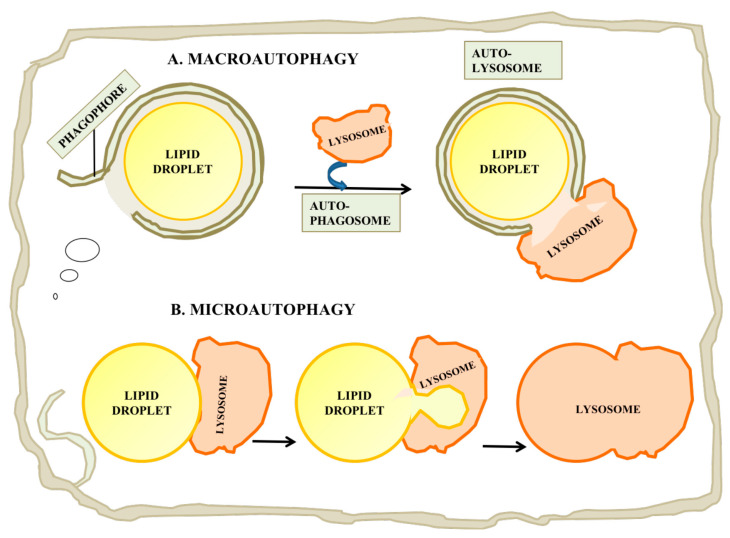
Catabolism of LDs by autophagy. (**A**) Macroautophagy is a process by which the entire LD can be enveloped by a phagophore or isolation membrane which seals to become an autophagosome. The autophagosome then undergoes a maturation process and fuses with a lysosome, leading to digestion of the LD and release of free fatty acid, glycerol and cholesterol. (**B**) Microautophagy of LDs has also been reported. While this process is not currently well understood, it may involve the internalization of a small portion of the LD into an internal vesicle in the lysosome. This process may allow autophagy of lipid from LDs which are too large for macroautophagy.

While cytoplasmic lipolytic enzymes play an important role in releasing FFA and free cholesterol, there is strong evidence for the importance of a parallel pathway involving lysosomal degradation of LDs (Figure 4A). Singh et al. provided the initial evidence for autophagy of LDs and coined the term “lipophagy” for this process [93], and further work by Ouimet [94] provided evidence that in a macrophage model of foam cell formation, inhibition of lipophagy largely blocked release of cholesterol from LDs, i.e., that in some cell types, lipophagy was more than a supplemental pathway and was critical to catabolism of neutral lipids. Extensive further studies of lipophagy have been made in a variety of systems, including: yeast, *Drosophila*, *C. elegans,* mammalian tissue culture and mammalian in vivo systems (reviewed in [95]).

Macroautophagy is a complex multi-step process for targeting cytoplasmic material including organelles to lysosomes. While macroautophagy is still not completely understood, large portions of the process have been characterized, starting with the initial identification of many of the key proteins through yeast genetics [96]. The process will only be summarized here, as there are many excellent reviews available (e.g., [96,97,98,99]). There are a variety of means for initiating macroautophagy, including inactivation of mammalian target of rapamycin complex 1 (mTORC1), which detects nutritional deprivation or, alternatively, organelles can be tagged for destruction by the covalent attachment of ubiquitin or other molecules. Macroautophagy is initiated by the Atg1/ULK complex upon inactivation of mTORC1. The Atg1 complex recruits Atg9 vesicles, which play a still poorly understood role in macroautophagy initiation, and a phosphoinositide 3-kinase (PI3K) complex [100]. Membrane-associated phosphatidylinositol 3-phosphate (PI(3)P) on the autophagosome precursor then recruits other proteins, which in a multi-step process expands and modifies the membrane of the autophagosome precursor, also known as a phagophore or an isolation membrane [100]. In a later step, Atg8-family proteins including LC3 are recruited to the isolation membrane [98]. LC3 can bind to a number of “autophagy receptors” including p62, which can bind ubiquitinated or otherwise tagged organelles and protein aggregates. Binding of autophagy receptors to organelles including mitochondria, and LDs to protein aggregates has been shown to favor macroautophagy and lysosomal degradation of these organelles [101]. In the case of mitochondria, this appears to be part of a quality control process which identifies and targets defective mitochondria for removal [102]. The isolation membrane can partially surround an organelle through interaction with autophagy receptors [98]. The initial steps of macroautophagy are completed when the phagophore membrane seals to form an autophagosome [98]. It then begins a process of maturation that includes the interaction with elements of the endocytic pathway dependent in part on Rab7 and Rab10 [103]. Finally, the modified autophagosome fuses with lysosomes in a Rab7-dependent process and its contents are degraded by the numerous enzymes present there. One enzyme, lysosomal acid lipase (LAL), is highly relevant to LDs as it can release both FFA and free cholesterol [104]. Other enzymes, including a wide range of proteases, glycases and lipases, enable the complete degradation of most of the biological polymers that can be encountered.

The important role of macroautophagy in the turnover of LDs and in mobilization of stored lipids was not appreciated until after the cytoplasmic enzymes had been characterized. Singh and coworkers [93] found that inhibition of macroautophagy in cultured hepatocytes and in mouse liver led to intracellular accumulation of TAG. This indicated that at least in some cell types, maintenance of heavy and continuing lipid metabolism required the action of lysosomal enzymes in addition to the cytoplasmic enzymes just discussed. Following this study, Ouimet [94] reported that efflux of cholesterol from monocytes depended on macroautophagy in a foam cell model. In this system, mouse monocytes were loaded with isotopically labeled cholesterol, which was then stored in LDs in the form of cholesterol esters. Efflux of the cholesterol to HDL would then depend on hydrolysis of LD-associated cholesterol esters. This cholesterol efflux was blocked by inhibitors of macroautophagy or of lysosomal acidification, but was unaffected by inhibitors of cytoplasmic neutral lipases. Furthermore, LDs were visualized to directly colocalize with lysosomes and with the macroautophagic marker LC3. Taken together, these studies showed that macroautophagy of LDs was an important pathway for neutral lipid catabolism and that in some circumstances it was the dominant pathway.

### 4.3. Rab18 and Macroautophagy

The small GTPase Rab18 has been implicated as a regulator of lipophagy. While Lutcke and coworkers [105] first reported Rab18 on apically localized endosomes in polarized cells shortly after the discovery of this Rab protein, a specific role for Rab18 in the endocytic pathway was first suggested only much later as a player in macroautophagy [106]. A number of subsequent studies then further implicated Rab18 in macroautophagy and provided evidence that part of the phenotype of Warburg micro syndrome in which Rab18 or its exchange factor RAB3GAP1/RAB3GAP2 is mutated [107,108] may be due to impaired macroautophagy. Warburg micro syndrome is a genetic disease characterized by a constellation of symptoms including: severe mental retardation, absence of the corpus callosum and cataracts, among others [109]. BasuRay reported that Rab18 knockdown increased autophagy flux in human stellate cells [110]. Nian and coworkers [111] reported that in neuronal cells from mice Rab18 could be cofractionated with late endosome/lysosome associated Rab7 and also colocalized with lysosomes by immunofluorescence. *Rab18 -/-* mice developed as a model of Warburg micro syndrome showed macroautophagy defects in this study [111].

A recent study suggests the molecular mechanism by which Rab18 could regulate macroautophagy. Takats and coworkers [112] used a *Drosophila* model system which provided evidence that Vps34 Complex I, which is an important protein complex involved in maturation of autophagosomes to lysosomes, and is an effector of Rab18. In this study, they reported a GTP-dependent interaction between Rab18 and the Atg6 component of the complex. Mutations in Rab18 or the Rab18 exchange factor RAB3GAP2 both resulted in the accumulation of immature autophagosomes which could be mimicked by knockdown of elements of the Vps34 Complex I, but not Vps34 Complex II (which is involved in endosome maturation but not macroautophagy). Another recent study [113] examined the effect of Rab18 knockout on macroautophagy in HeLa cells. This study found that basal macroautophagy could proceed normally after Rab18 knockout, but that starvation-induced macroautophagy was impaired. Taken together, these studies strongly implicate Rab18 as a regulator of macroautophagy and suggest that the phenotype of Warburg micro syndrome is related in part to dysfunctional macroautophagy due to impaired Rab18 function.

### 4.4. Effect of LD Size on Macroautophagy

In mammalian cells, LDs are often larger than lysosomes, potentially complicating autophagy. Schott [114] provided evidence that in mammalian hepatocytes ATGL was preferentially associated with large LDs, while catabolism of smaller LDs proceeded primarily by lipophagy. In this study, they proposed a division of labor in which large LDs were reduced in size through the action of lipolytic enzymes [114]. Such LDs, after reduction in size, would primarily be consumed by macroautophagy in their model, together with nascent LDs that have not yet become too large. It should also be pointed out that there are distinct regulatory pathways for macroautophagy and for activation of cytoplasmic enzymes. It would not be surprising if the division of labor between the two could vary in a cell-type and circumstance-dependent manner.

### 4.5. Microautophagy

While most studies have concentrated on the roles of macroautophagy in LD catabolism, in which a LD is engulfed in toto, some recent reports have suggested a complementary role for a form of microautophagy in which a portion of a LD’s complement of lipid is drawn into a lysosome and hydrolyzed (Figure 4B; reviewed in [115]). The process in yeast is believed to be independent of the conventional autophagy pathway, but possibly dependent on endosomal sorting complex required for transport (ESCRT) proteins, which are required to drive internal vesiculation into lysosomes, on seipin complex partners and on Niemann-Pick-type C protein homologues. There are several reports of stress-induced microautophagy of LDs in yeast (e.g., [116]). However until recently it was unclear whether a similar process could occur in mammalian cells, in which lysosomes are smaller relative to LDs than the yeast vacuole, which is typically larger than yeast LDs [115]. Schultze and coworkers have recently reported a microautophagy-like process in a murine hepatocyte cell line [117]. In this study, they identified transient (30 s to several min) interactions between lysosomes and LDs by time-lapse imaging of living cells. This process appeared to be independent of the conventional autophagy machinery, as it was independent of a number of factors required for macroautophagy including Atg5, Atg2, Rab7 and Rab10. Electron microscopy showed tight interactions between LDs and lysosomes in which portions of the LDs attached to the lysosomes were distorted and appeared to penetrate into the lysosome, suggesting some sort of force applied to the LD surface. The mechanism by which lipids were transferred to the lysosome was unclear, but the authors suggested the possibility that hemifusion between the LD and the lysosome would facilitate direct exposure of LD materials to lysosomal enzymes [117].

## 5. Concluding Remarks

While LDs are superficially very different from the membrane-bound intracellular organelles that have historically been considered to constitute secretory and endocytic pathways, their biogenesis and ultimate fate is intimately intertwined with both pathways. Existing evidence indicates that LDs originate from ER membranes through a process that involves phase separation of lipids with an important contribution of regulatory proteins. They can then remain associated with the ER through direct membranous connections, which can allow the diffusion of lipids and a select set of proteins between ER and the LD, or alternatively through protein tethers, or they can more completely but reversibly separate from the ER. The mature LD is also interacting with cytoplasmic factors including lipolytic enzymes, which can release fatty acids and cholesterol when needed. During this phase it can maintain or re-establish connections with the ER although the regulation of this process is still poorly understood. At the end of its life cycle, the LD will interact with elements of the endocytic pathway through both macroautophagy and microphagy. Thus, what was historically considered a static depot of fat is in fact a full member of the secretory/endocytic pathways.
